# Postharvest Spectral Light Composition Affects Chilling Injury in Anthurium Cut Flowers

**DOI:** 10.3389/fpls.2020.00846

**Published:** 2020-06-12

**Authors:** Sasan Aliniaeifard, Zahra Falahi, Shirin Dianati Daylami, Tao Li, Ernst Woltering

**Affiliations:** ^1^Photosynthesis Laboratory, Department of Horticulture, Aburaihan Campus, University of Tehran, Tehran, Iran; ^2^Institute of Environment and Sustainable Development in Agriculture, Chinese Academy of Agricultural Sciences, Beijing, China; ^3^Wageningen Food & Biobased Research, Wageningen, Netherlands; ^4^Horticulture & Product Physiology Group, Department of Plant Sciences, Wageningen University, Wageningen, Netherlands

**Keywords:** anthurium, water loss, transpiration rate, chilling stress, light spectrum

## Abstract

The effect of the lighting environment during postharvest storage of ornamentals has largely been neglected in previous research. Anthurium is a cold-sensitive species originating from tropical climates and is widely cultivated all around the world for its colorful spathes. To investigate the effects of light spectrum on the performance of Anthurium cut flowers under cold storage, two cultivars [Calore (red spathe) and Angel (withe spathe)] were placed at low temperature (4°C), either in darkness (D) or under different light spectra [red (R), blue (B), 70:30% red:blue (RB), and white (W)] at an intensity of 125 µmol.m^−2^.s^−1^. In both cultivars, the longest and shortest vase lives were observed in spathes exposed to the R and B spectra, respectively. In both cultivars, electrolyte leakage (EL) of spathe was highest under the B and W spectra and lowest under the R spectrum. The highest rate of flower water loss from the spathes was observed under the B-containing light spectra, whereas the lowest rate of water loss was observed in D and under the R spectrum. Negative correlations were observed between EL and vase life and between anthocyanin concentration and EL for both Anthurium cultivars. A positive correlation was found between anthocyanin concentration and vase life. For both Anthurium cultivars, spectral light composition with higher percentage of B resulted in higher EL and as a result shorter vase life in cut flowers under cold storage condition. The negative effect of the B light spectrum on vase life of Anthurium can be explained through its effect on water loss and on oxidative stress and membrane integrity. The quality of Anthurium cut flowers should benefit from environments with restricted B light spectrum during postharvest handling.

## Introduction

Tropical flowers are produced worldwide because of their ornamental values. Postharvest handling of tropical flowers (*e.g.* Anthurium) is usually difficult due to their sensitivity to low temperatures. Anthurium (*Anthurium andraeanum*) is a tropical plant used in ornamental industry for its colorful spathes and green leaves. It is produced in wide ranges of climates; in locations far away from their original habitats in greenhouses. Although cut Anthurium has a long vase life when compared to the vase life of many other cut flowers (Mujaffar and Sankat, 2003), postharvest exposure to low temperatures can reduce its vase life. Temperatures below 12°C induce symptoms of chilling injury such as brown spots on the Anthurium spathe (Promyou et al., 2012). In the winter time of temperate and cold climates, to prevent negative effects of cold temperatures on Anthurium cut flowers, the growers transfer the flowers to higher temperatures immediately after harvest. The importance of chilling stress for Anthurium flowers can be highlighted during their transport, storage, and distribution in the winter time. Anthurium flowers may be exposed to low temperatures as they are often part of mixed transport or storage with other flowers that need lower temperatures. This may cause a decrease in the quality of the spathe in customer locations. Browning and blueing of spathe and wilting of spadix are observed in Anthurium cut flowers when they face temperatures lower than 12°C (Promyou et al., 2012; Soleimani Aghdam et al., 2015).

Much effort has been made during the last decade to decrease the susceptibility of Anthurium cut flowers to low temperatures (Promyou et al., 2012; Soleimani Aghdam et al., 2015; Soleimani Aghdam et al., 2016b). These efforts were mainly focused on the application of chemical solutions to the stems of cut flowers. For instance, salicylic acid (Promyou et al., 2012) and *γ*-aminobutyric acid (GABA) (Soleimani Aghdam et al., 2015; Soleimani Aghdam et al., 2016b; Soleimani Aghdam et al., 2016c) have successfully decreased the negative effects of low temperatures on cut flower quality and vase life. Application of these chemicals caused approximately 10% reduction in occurrence of browning of Anthurium spathes. In the other cases no effect of chemical solutions was observed. For instance, using calcium in holding solution did not result in vase life improvement in cold-stored Anthurium cut flowers (Ketrodsakul et al., 2016). There is still a need for preventing the problems related to chilling injury beyond the use of chemicals.

In some cut flowers with long vase life such as Anthurium, long distance transport to customers is common. During the postharvest period the cut flowers are usually held in darkness and may be exposed to cold (<12°C). A positive role for the presence of light during postharvest storage has been proposed for keeping quality of flowers with photosynthetic leaves such as rose, chrysanthemum, and protea (Rajapakse and Kelly, 1994; Ranwala and Miller, 2000). To the best of our knowledge, no study has been done on the effect of light (spectra) on postharvest quality of cut flowers such as Anthurium.

Nowadays, light emitting diodes (LEDs) lighting technology is widely used in horticulture (Kozai et al., 2015). The LEDs have long lifetimes, small mass, low heat production, high radiant efficiency, physical robustness, and narrow spectrum (Tennessen et al., 1994; Kim et al., 2004; Breive et al., 2005; Morrow, 2008). By using LEDs it is possible to apply specific wavelengths to plant material to study particular plant responses. Red (R) and blue (B) wavelengths are the main light spectra influencing water relations and gas exchange features of the plants (Massa et al., 2008; Hogewoning et al., 2010; Ouzounis et al., 2014; Ouzounis et al., 2016). Research on the effect of spectral wavelengths on plant responses is still ongoing. After harvest, cut flowers still respond to environmental cues such as light spectra. Many plant processes are influenced by light spectrum in the range between 380 and 750 nm. For instance, B light induces water influx into the guard cells and thus, stomatal opening through activation of plasma membrane H^+^-ATPases (Kinoshita and Hayashi, 2011). Red light increases phosphorylation levels of the H^+^-ATPase in response to the B light and therefore has inductive effects on B light-dependent stomatal opening and water loss by the leaf (Olsen et al., 2002; Suetsugu et al., 2014).

The importance of spathe water loss during postharvest storage under different temperatures has been previously reported (Sankat and Mujaffar, 1994). It was shown that at temperatures above 13°C when transpiration is more than the water uptake; the product would not be marketable anymore. At 8°C both water uptake and transpiration will be hampered and chilling symptoms (*e.g.* blueing and browning of the spathes) will appear (Sankat and Mujaffar, 1994).

Spectrum is one of the light characteristics that considerably influence plant responses. Light quality not only influences photosynthesis but also cellular integrity, water relations, pigmentation, carbohydrate, and antioxidant status of the plants (Bayat et al., 2018; Hosseini et al., 2018; Hosseini et al., 2019). We hypothesized that different light spectra through their effects on cellular metabolism can influence the cold tolerance and quality of the spathe and hence the vase life of the Anthurium flower. The main aim of this study was to investigate the effects of different light spectra on postharvest performance of Anthurium cut flowers at a chilling temperature.

## Materials and Methods

### Flowers and Lighting Treatments

Cut flowers of Anthurium (*Anthurium andraeanum*) cultivars with red (‘Calore’) and white (‘Angel’) spathes were obtained from a commercial Anthurium greenhouse in the morning. Both cultivars were said to have long vase life in temperatures over 13°C, but ‘Angel’ was considered more sensitive to cold than ‘Calore’ (personal communication with Anthurium growers).

Anthurium cut flowers were harvested at the commercial harvest stage when 40–50% of the spadix true flowers were fully opened (Promyou et al., 2012). In the greenhouse, flowers with no deformities, no bruises, and straight peduncles were cut and placed into 50 ml tubes containing water. They were immediately transported (within less than 1 h after harvest) at 21°C to the laboratory where the flower stems were recut to 30 cm length. Each flower was placed in flasks containing 500 ml tap water. Sixty flasks with cut flowers (30 cut flowers from each cultivar) were placed into the light treatment compartments (l × w × h = 0.8 m × 0.5m × 0.5m). Compartments were positioned in a climate room with relative humidity of 80–81% and fixed temperature of 4°C provided by constant ventilation inside the cold rooms.

**Figure 1:**
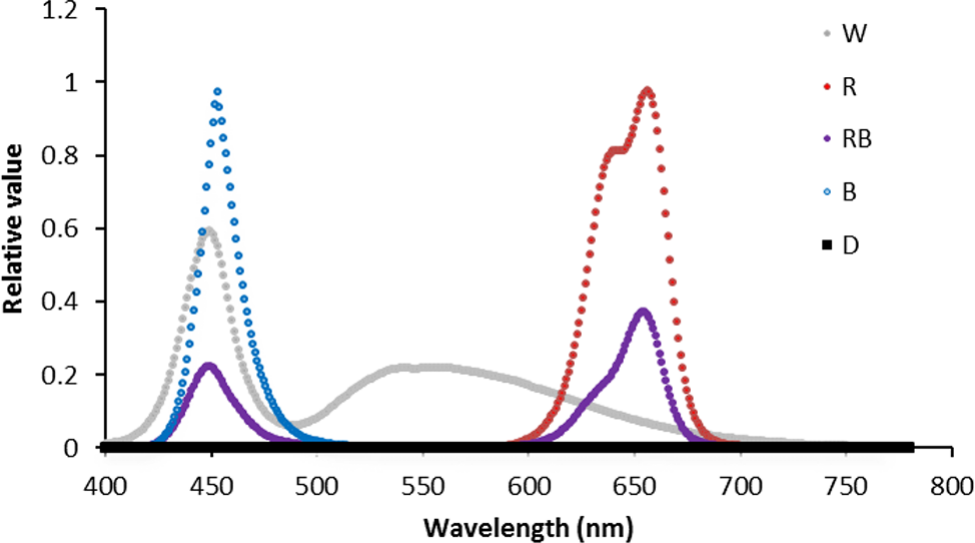
Light spectra of white (W), red (R), red and blue (RB), blue (B), and dark (D) lighting environments measured at spathe level in the chambers at 4°C.

Twelve flasks (six per each cultivar) were placed in each of the aforementioned compartments in the dark (D) and under continuous light intensity of 125 ± 5 μmol m^−2^ s^−1^ photosynthetic photon flux density (PPFD) but with different light spectra including white (W), blue (B), red (R), and 70% R + 30% B (RB) provided by LED production modules (24W, Iran Grow Light Co, Iran). Light intensity and spectra were measured using a light meter (Sekonic C-7000, Japan) (**Figure 1**).

### Visual Quality and Vase Life

Each flower was inspected daily, and its vase life was estimated based on the visual symptoms such as loss of spathe glossiness, desiccation of spadix, blackening and wilting of spathe (Paull and Goo, 1982) as well as browning and desiccation of spadix. Visual symptoms on the spathes were recorded using a scale from 1 to 5; 1 = no chilling injury; 2 = mild injury (1–20% of spathe affected); 3 = moderate injury (21–50% of spathe affected); 4 = severe injury (51–80% of spathe affected); 5 = very severe injury (81–100% of spathe affected).

### Relative Water Loss Percentage

Every day during the experiment, fresh weight (FW) of individual Anthurium cut flower was measured. FW differences between two subsequent days were calculated (ΔFW). Accumulative water loss of the Anthurium cut flowers was expressed as percentage water loss relative to the initial weight.

### Electrolyte Leakage

For measuring EL, 20 discs (8 mm) per spathe (three spathes) were floated in 10 ml deionized water in closed vials and incubated at 25°C on a rotary shaker for 24 h. Thereafter, electrical conductivity of the solution (C_1_) was determined using a conductivity meter (Metrohm, Switzerland). Subsequently, samples were autoclaved at 120°C for 20 min, and the electrical conductivities of the solution (C_2_) were again recorded after equilibration at 25°C. EL was calculated based on the following equation.

$$\mathrm{EL}=\frac{C_{1}}{C_{2}}\times 100$$

### Osmotic Potential

Osmotic potential of the spathe was determined using the method described by Martìnez et al. (2004). The measurement was performed on three flowers per treatment. Spathes were cut into small segments; tissue pieces were placed in Eppendorf tubes perforated with four small holes and immediately frozen in liquid nitrogen. After being individually encased in a second intact Eppendorf tube, the samples were allowed to thaw for 30 min and centrifuged at 15,000 g for 15 min at 4°C. The extracted sap was collected and used for *ψ*s determination. Osmolarity (c) was measured with a vapor pressure osmometer (Osmomat 030-gonatec) and converted from mOsmole kg^−1^ to MPa according to the Van’t Hoff equation (Martìnez et al., 2004).

### Proline Concentration

Free proline content was measured based on the method described by Bates et al. (1973). This measurement was performed on three spathes per treatment. Grounded spathe samples (0.5 g) were homogenized in 3% (w/v) sulphosalicylic acid and then filtered through filter papers. Following addition of acid-ninhydrin and glacial acetic acid, the mixture was heated at 100°C for 1 h in a water bath. Reaction was stopped using an ice bath. The mixture was extracted with toluene, and the absorbance of the fraction with toluene aspired from the liquid phase was read at 520 nm (Perkin Elmer Lambda 25 UV-VIS Spectrometer). Proline concentration was determined using a calibration curve and expressed as μmol proline g^−1^ FW (Bates et al., 1973).

### Hydrogen Peroxide

Hydrogen peroxide (H_2_O_2_) content was spectrophotometrically (Perkin Elmer Lambda 25 UV-VIS Spectrometer) measured after reaction with potassium iodide (KI). Ground spathe samples (0.25 g) were homogenized in 0.1% trichloroacetic acid (TCA) and centrifuged at 5,000 g for 10 min. The reaction mixture contained 0.5 ml of 0.1% trichloroacetic acid (TCA), spathe extract supernatant, 0.5 ml of 100 mm K-phosphate buffer and 2 ml reagent (1 m KI w/v in fresh double-distilled H_2_O). The blank contained 0.1% TCA in the absence of leaf extract. The reaction was developed for 1 h in darkness, and absorbance was measured at 390 nm. The amount of H_2_O_2_ was calculated using a standard curve prepared with known concentrations of H_2_O_2_ according to the method described by Patterson et al. (1984). This measurement was performed on three spathes per treatment.

### Carbohydrate Contents

Ground spathe samples (300 mg FW) were mixed with 7 ml of 70% ethanol (w/v) for 5 min on ice and centrifuged at 6,700 *g* for 10 min at 4°C. After adding 200 ml of the supernatant to 1 ml of an anthrone solution (0.5 g anthrone, 250 ml 95% H_2_SO_4_, and 12.5 ml distilled water), the absorbance was spectrophotometrically (Perkin Elmer Lambda 25 UV-VIS Spectrometer) recorded at 625 nm (van Doorn, 2012). This measurement was performed on three spathes per treatment.

### Pigments

The Chl and carotenoid contents of the spathes were measured according to the method described by Arnon (1949). For measuring the anthocyanin content of the spathes, 1 g of ground spathe tissue was homogenized in 10 ml methanol, and the extract was incubated at 4°C in the dark overnight. The slurry was centrifuged (SIGMA-3K30) at 4,000 *g* for 10 min. The anthocyanin in the supernatant was spectrophotometrically (Perkin Elmer Lambda 25 UV-VIS Spectrometer) determined at 520 nm according to the method described by Wagner (1979). This measurement was performed on three spathes per treatment.

### Statistical Analysis

Nondestructive measurements (water loss and vase life) were done on six spathes as six replicates. Destructive measurements were done on three spathes as three biological replicates. The experiment was repeated twice in precisely controlled cold room with fixed temperature of 4°C. Since the results of both experiments for the vase life were identical between the two experiments, part of the destructive measurements was performed in flowers from the first experiment and part of the measurements in flowers from the repeated experiment. In both experiments visual judgement of spathe quality and daily water loss were measured. Apart from these measurements, in the first experiment, EL, chlorophyll and carotenoids content, photosynthetic activity, and spathe characteristics were determined; in the second experiment osmotic potential, carbohydrates, anthocyanins, proline and hydrogen peroxide contents were determined after 14 days of treatment. Osmotic potential and EL were determined in fresh spathe samples; the other analyses were done in spathe samples ground in liquid nitrogen and kept in −80°C. In all the cases measurements were performed in three or more biological replications.

The data were subjected to two-way analysis of variance (ANOVA), and Tukey was used as a means separation test, and P > 0.01 was considered not significant. The percentage of spathe water loss data was fitted with linear regression, and F test was used for comparing the slopes of the curves. GraphPad Prism 7.01 for Windows (GraphPad software, Inc. San Diego, CA) was used for statistical analysis and comparisons among treatments.

## Results

### Vase Life of Anthurium Depends on Postharvest Light Spectra and Cultivar

**Figure 2:**
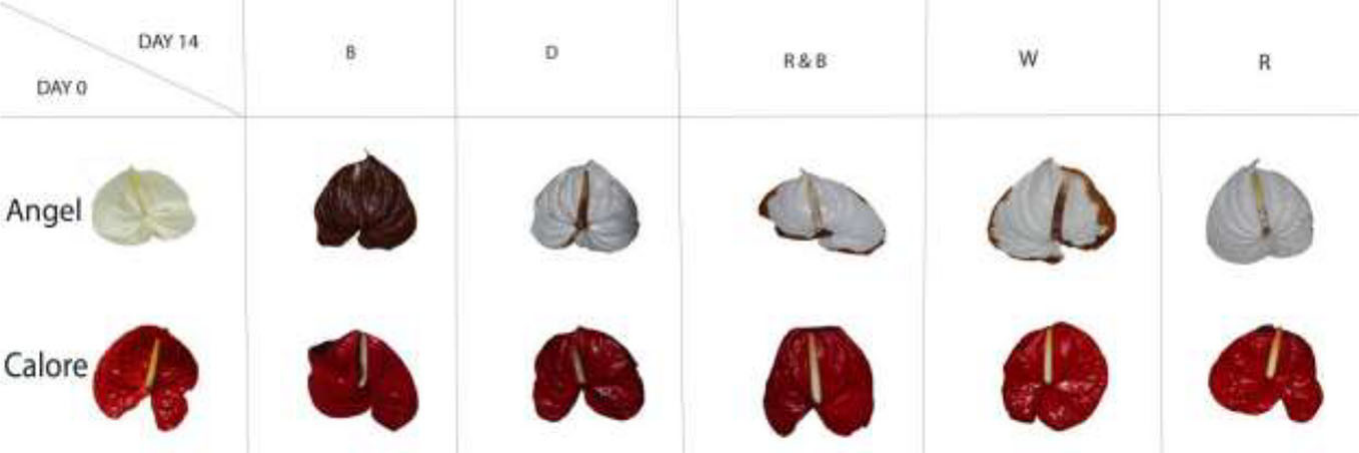
Chilling injury symptoms in Anthurium cut flowers (‘Angel’ and ‘Calore’) held in the dark (D) or continuously exposed to 125 ± 5 μmol m^−2^ s^−1^ of different light spectra [white (W), red (R), red and blue (RB), blue (B)] after 14 days vase life at 4°C.

**Figure 3:**
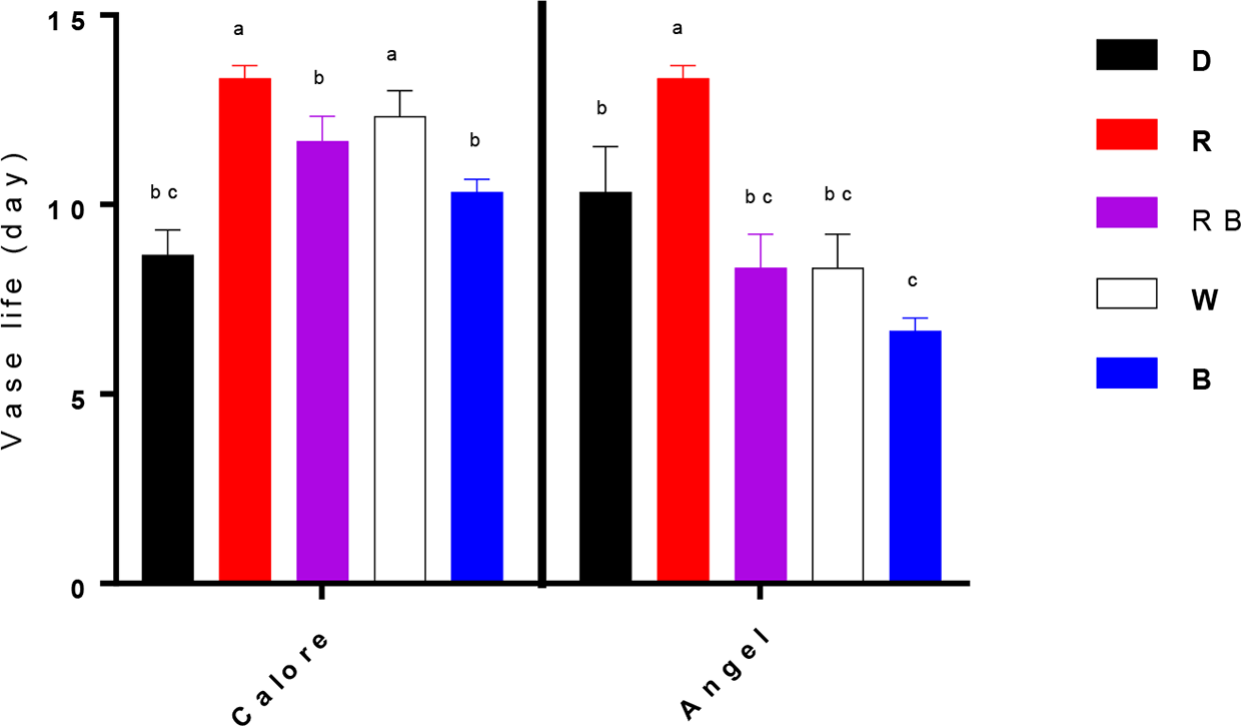
Vase life of Anthurium cut flowers (‘Angel’ and ‘Calore’) held in the dark (D) or continuously exposed to 125 ± 5 μmol m^−2^ s^−1^ of different light spectra [white (W), red (R), red and blue (RB), blue (B)] at 4°C. End of vase life was determined by visual symptoms such as loss of spathe glossiness, desiccation of spadix, blackening and wilting of spathe as well as browning and desiccation of spadix. Vase life was terminated when flowers rated 3 for spathe browning or 4 for spadix necrosis. Values are the means of six biological replicates, and bars indicate means ± SEM. Bars with different letters are significantly different (ANOVA, P < 0.01).

Vase life of Anthurium cut flowers at 4°C was significantly (P ≤ 0.01, **Table 1**) influenced by the interaction between light spectra and cultivar (**Figures 2** and **3**). Among the light spectra, the longest vase life was observed in spathes exposed to R in both cultivars (**Figure 3**). In ‘Angel’, the shortest vase life was observed under W, B, and RB spectra (**Figures 2** and **3**). After 14 days exposure to B spectrum, the spathe and spadix of ‘Angel’ had become dark brown (**Figure 2**). In ‘Calore’, exposure to D, B, and RB spectra resulted in shorter vase life when compared to the vase life of flowers exposed to R and W spectra. These results indicate that R spectrum is able to prolong the vase life of Anthurium cut flowers in cold environments and that vase life under light was roughly dependent on the percentage of B wavelengths in the overall spectrum: the more B, the shorter the vase life.

**Table 1:** Analysis of variance (*F* values) for assessed parameters for Anthurium cut flowers exposed to different light spectra under cold storage condition (4°C).

	Independent variables		
Dependent Variable	Light spectra	Cultivar	Interaction (Spectra × Cultivar)
Vase life	13.8^**^	18.2^**^	6.8^**^
EL	7.01^***^	57.6^**^	13.1^**^
Osmotic potential	4.5^***^	13.1^***^	1.4^ns^
Proline	3.8^*^	71.1^**^	6.7^**^
Anthocyanin	17^****^	45^****^	4.7^**^
Carotenoids	0.55^ns^	0.29^ns^	0.54^ns^
Soluble carbohydrates	2.9^*^	0.35^ns^	13.8^**^
Hydrogen peroxide	16.01^****^	296^****^	17.39^****^

ns, Nonsignificance. *Significance at 0.05 probability level. ^**^Significance at 0.01 probability level. ^***^Significance at 0.001 probability level. ^****^Significance at 0.0001 probability level.

### Spathe Water Loss Is Controlled by Postharvest Light Spectra

**Figure 4:**
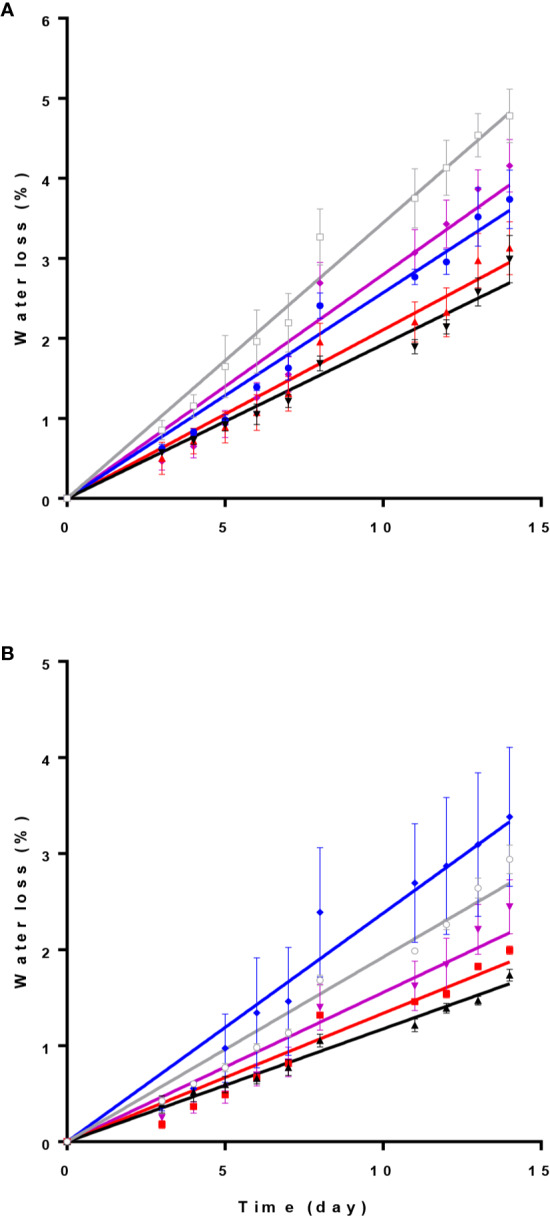
Relative water loss percentage in Anthurium cut flowers [‘Calore’ **(A)** and ‘Angel’ **(B)**] held in the dark (D) or continuously exposed to 125 ± 5 μmol m^−2^ s^−1^ of different light spectra [white (W), red (R), red and blue (RB), blue (B)] during 14 days storage at 4^°^C. Values are the means of six biological replicates, and bars indicate means ± SEM. Data were fitted with linear regression, and the lines indicate the fitted line. Color of each line corresponds to the light spectrum of each treatment, gray stand for W spectrum.

Relative water loss percentage during 14 days of experiment was lowest under D and R spectrum in both cultivars (**Figure 4**). In ‘Calore’ (**Figure 4A**), the highest relative water loss percentage was observed under W; in ‘Angel’ under B spectrum (**Figure 4B**). The slope of the water loss curve under W light in ‘Calore’ and under B lights in ‘Angel’ was approximately doubled in comparison with the slope of the curves in D (**Table 2**). In both cultivars, no statistical differences for the slope of water loss were observed between D and R spectrum.

**Table 2:** Slope of the curve for accumulative percentage of water loss over time and spathe osmotic potential after 14 days of treatment in two Anthurium cut flowers (‘Angel’ and ‘Calore’) exposed to different light spectra [white (W), red (R), red and blue (RB), blue (B) and dark (D)] at 4°C.

Cultivar	Light spectrum	Slope	Osmotic potential (Mpa)
	**W**	0.34 ± 0.007 ^a^	−1.41 ± 0.32^A,b^
	**D**	0.19 ± 0.005 ^e^	−1.71 ± 0.27^A,ab^
Calore	**R**	0.21 ± 0.006 ^de^	−1.13 ± 0.33^A,b^
	**B**	0.26 ± 0.007 ^bc^	−2.02 ± 0.12^A,a^
	**RB**	0.28 ± 0.012 ^b^	−1.03 ± 0.25^A,b^
	**W**	0.19 ± 0.006 ^e^	−1.34 ± 0.02^B,a^
	**D**	0.12 ± 0.002 ^g^	−0.91 ± 0.05^B,b^
Angel	**R**	0.13 ± 0.005 ^g^	−0.82 ± 0.13^B,b^
	**B**	0.24 ± 0.005 ^c^	−1.21 ± 0.08^B,a^
	**RB**	0.15 ± 0.007 ^f^	−0.71 ± 0.12^B,b^

Values are the means of six biological replicates for the Slope and three biological replicates for the Osmotic potential, and bars indicate means ± SEM.

In the case of osmotic potential, different capital letters indicate the significant differences between cultivars and different small letters indicate significant difference among light spectra.

### Cellular Integrity and Osmoregulation Is Influenced by Light Spectra and Cultivar

**Figure 5:**
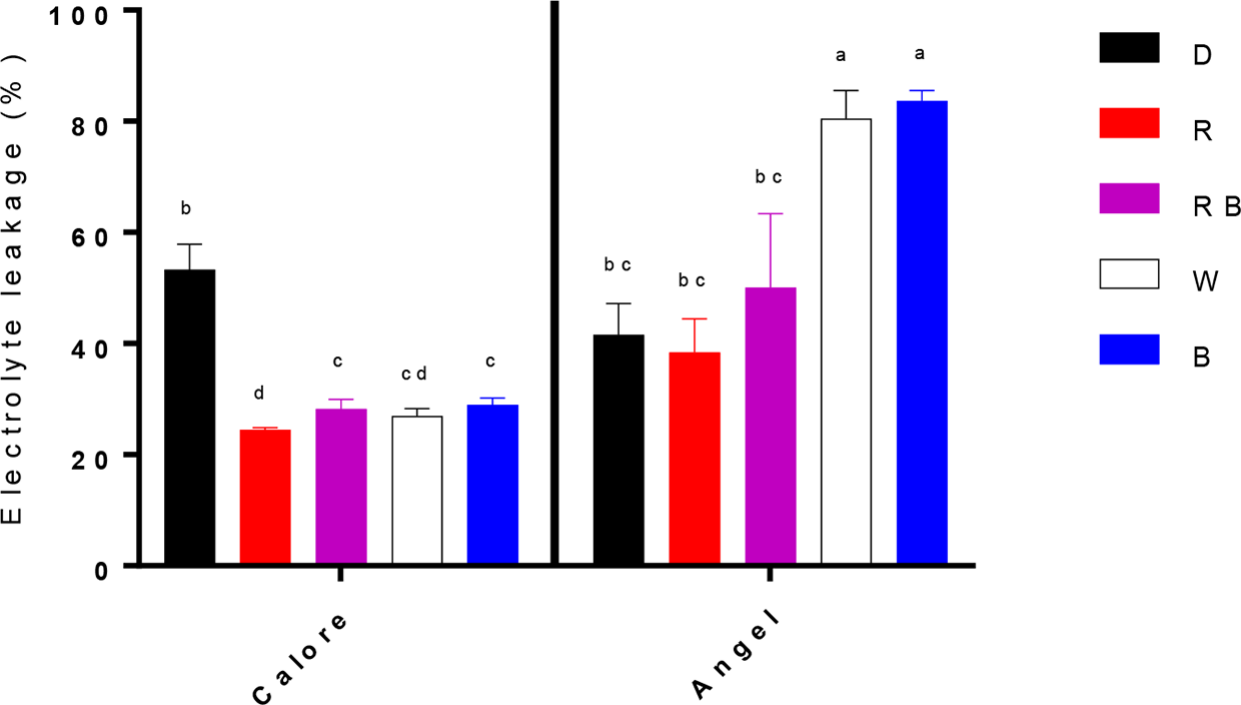
Electrolyte leakage from two cultivars of Anthurium cut flowers (‘Calore’ and ‘Angel’) held in the dark (D) or continuously exposed to 125 ± 5 μmol m^−2^ s^−1^ of different light spectra [white (W), red (R), red and blue (RB), blue (B)] following 14 days storage at 4°C. Values are the means of three biological replicates, and bars indicate means ± SEM. Bars with different letters are significantly different (ANOVA, P < 0.01).

Electrolyte leakage (EL) after 14 days of treatment was considerably influenced by the interaction between the light spectra and cultivar (P ≤ 0.01, **Table 1**). EL was generally higher in ‘Angel’ than in the ‘Calore’. In ‘Calore’, EL was significantly lower under all light treatments compared to D. The lowest EL in ‘Calore’ was detected in spathes exposed to the R spectrum. In ‘Angel’ EL was highest in spathes exposed to W and B spectra and was lowest in spathes exposed to R spectrum (**Figure 5**).

Osmotic potential was influenced by the single effects of the light spectra (P ≤ 0.001, **Table 1**) and cultivar (P ≤ 0.001, **Table 1**). Osmotic potential of the spathe in ‘Calore’ after 14 days of treatment was 32% higher (more negative) than osmotic potential of spathe in ‘Angel’ (**Table 2**). Among the light spectra, osmotic potential of ‘Calore’ spathes under the B spectrum was the highest. Under the B spectrum, osmotic potential was 40% higher than the osmotic potential under the R spectrum. In ‘Angel’ osmotic potential was highest under the W and B spectra (**Table 2**).

**Figure 6:**
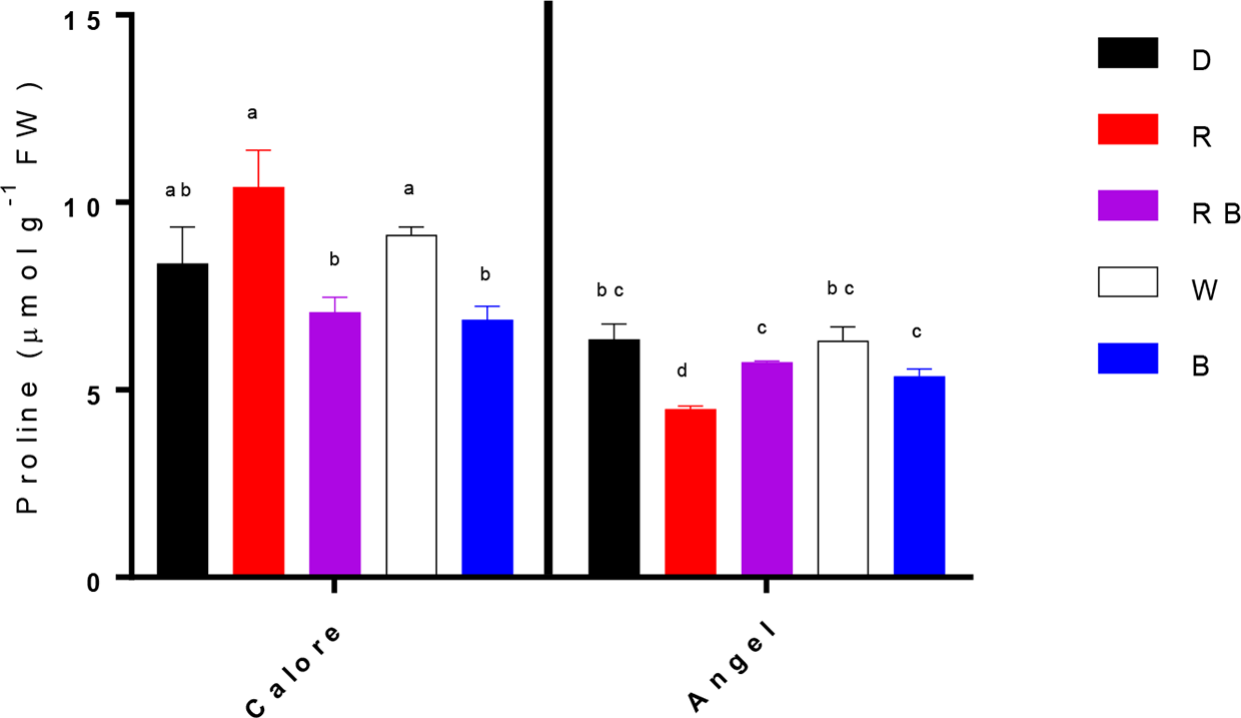
Proline contents in the spathes of two cultivars of Anthurium cut flowers (‘Calore’ and ‘Angel’) held in the dark (D) or continuously exposed to 125 ± 5 μmol m^−2^ s^−1^ of different light spectra [white (W), red (R), red and blue (RB), blue (B)] following 14 days storage at 4^°^C. Values are the means of three biological replicates, and bars indicate means ± SEM. Bars with different letters are significantly different (ANOVA, P < 0.01).

Under all light spectra, proline concentration in the spathes of ‘Calore’ was higher than in ‘Angel’ (**Figure 6**). In the spathe of ‘Calore’ the highest proline concentration was detected under the R spectrum; in ‘Angel’ the lowest proline concentration was measured under R light. There was no clear trend in proline concentration with the proportion of B spectrum.

**Figure 7:**
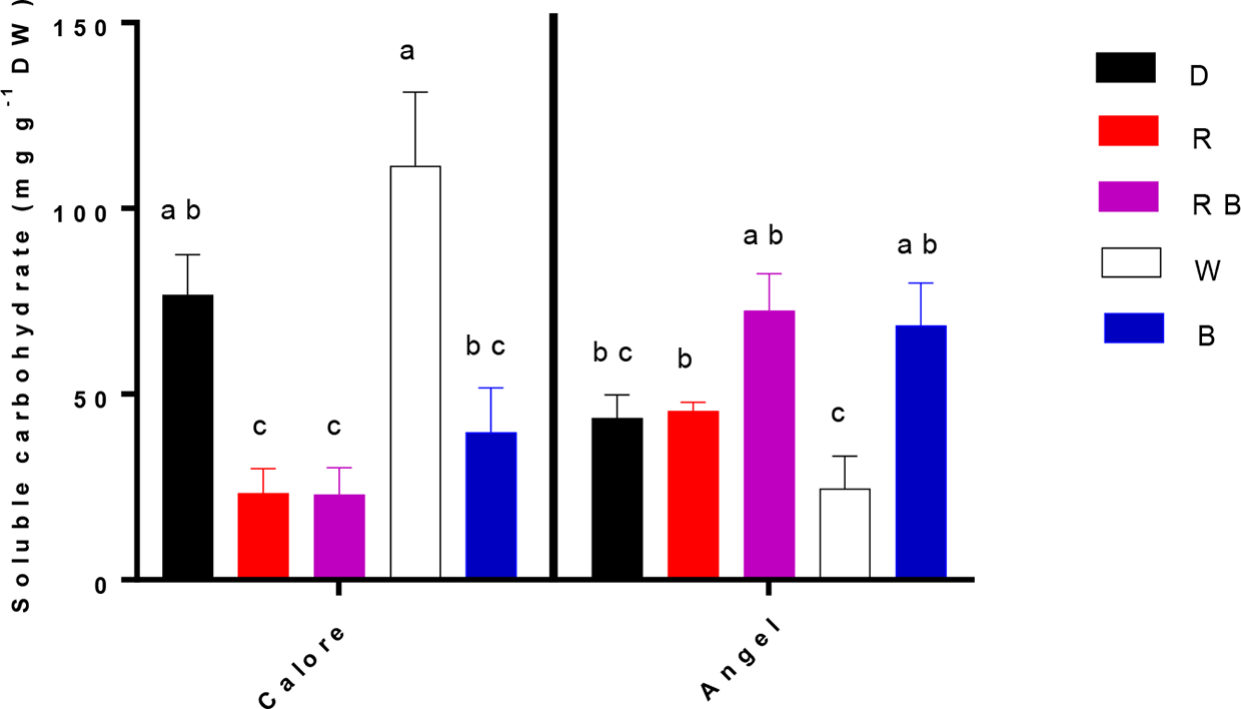
Soluble carbohydrate concentrations in the spathes of two cultivars of Anthurium cut flowers (‘Calore’ and ‘Angel’) held in the dark (D) or continuously exposed to 125 ± 5 μmol m^−2^ s^−1^ of different light spectra [white (W), red (R), red and blue (RB), blue (B)] following 14 days vase life at 4°C. Values are the means of three biological replicates, and bars indicate means ± SEM. Bars with different letters are significantly different (ANOVA, P < 0.01).

Concentration of soluble carbohydrates in the spathe was affected by the interaction between light spectra and cultivar (P ≤ 0.01, **Table 1**). In ‘Calore’, the lowest concentration of soluble carbohydrates was detected under the R, B, and RB spectra; the highest concentration of carbohydrates was detected under the W spectrum and D. In ‘Angel’, the concentration of soluble carbohydrates in W was lower than in the other treatments (**Figure 7**). There was no clear trend in carbohydrate concentration with the proportion of B spectrum.

**Figure 8:**
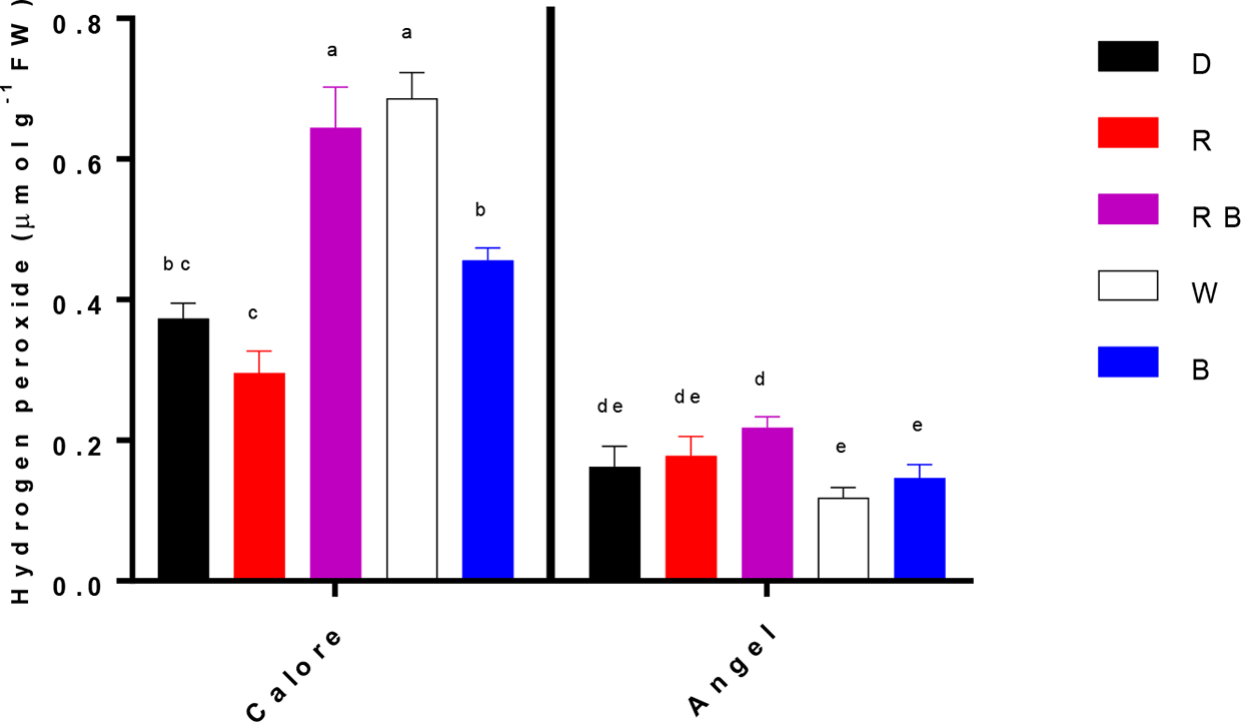
Hydrogen peroxide content in the spathes of two cultivars of Anthurium cut flowers (‘Calore’ and ‘Angel’) held in the dark (D) or continuously exposed to 125 ± 5 μmol m^−2^ s^v1^ of different light spectra [white (W), red (R), red and blue (RB), blue (B)] following 14 days vase life at 4°C. Values are the means of three biological replicates, and bars indicate means ± SEM. Bars with different letters are significantly different (ANOVA, P < 0.01).

Hydrogen peroxide content of the spathe was influenced by the interaction between light spectra and cultivar (P ≤ 0.00001, **Table 1**). Under all light spectra, higher hydrogen peroxide content was observed in ‘Calore’ in comparison with its content in ‘Angel’. In ‘Calore’, the highest hydrogen peroxide content was detected under the W and RB spectra. The hydrogen peroxide content of ‘Calore’ spathes was respectively six and three times higher than its content in ‘Angel’ spathes under the W and RB spectra (**Figure 8**).

### Spathe Anthocyanin Content Depends on Cultivar and Light Spectra

**Figure 9:**
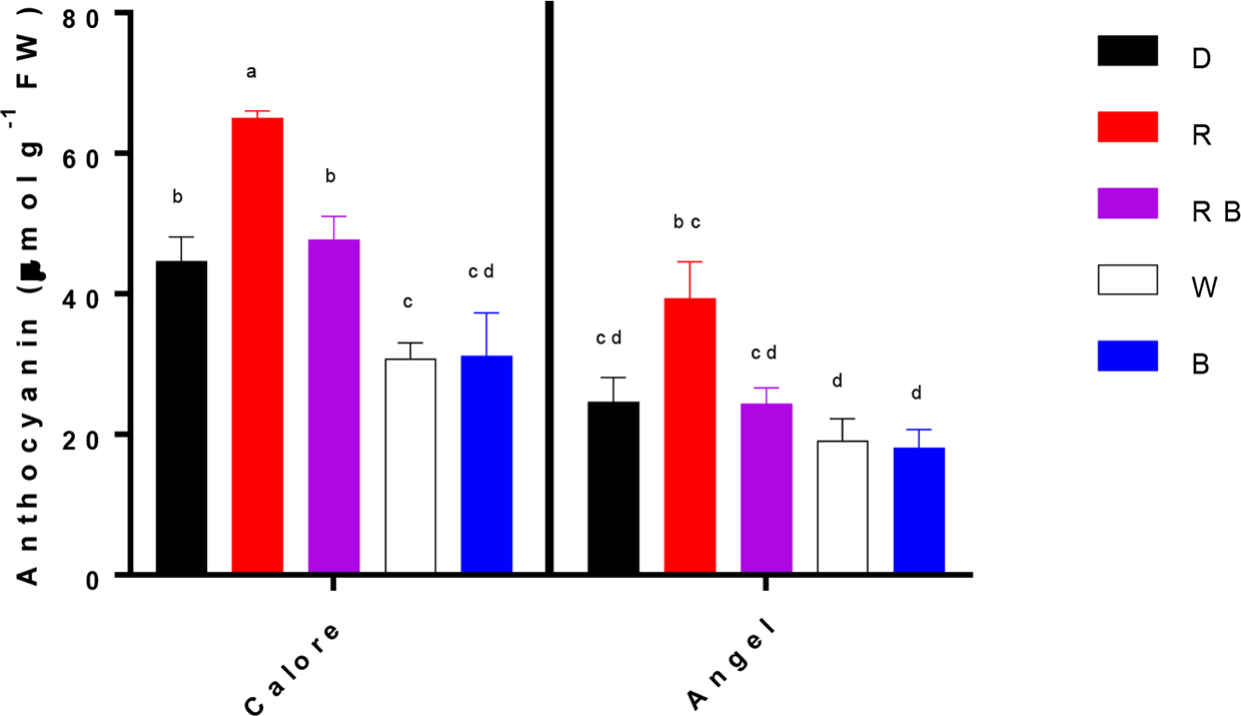
Anthocyanin concentrations in the spathes of two cultivars of Anthurium cut flowers (‘Calore’ and ‘Angel’) held in the dark (D) or continuously exposed to 125 ± 5 μmol m^−2^ s^−1^ of different light spectra [white (W), red (R), red and blue (RB), blue (B)] following 14 days vase life at 4°C. Values are the means of three biological replicates, and bars indicate means ± SEM. Bars with different letters are significantly different (ANOVA, P < 0.01).

Chlorophyll was not detected in the two studied Anthurium cultivars. No significant difference in carotenoid content was detected between the two cultivars or among the light spectra and their interactions (**Table 1**). Anthocyanin content of the spathe was affected by the interaction between light spectra and cultivar (P ≤ 0.01, **Table 1**). Under all light spectra, except B, higher anthocyanin concentration was detected in ‘Calore’ in comparison with ‘Angel’. In ‘Calore’, the highest anthocyanin concentration was detected under R spectrum; the lowest concentration was found under W and B spectra. Similarly, in ‘Angel’, the lowest anthocyanin concentration was detected under W and B spectra (**Figure 9**).

### Vase Life of Anthurium Depended on Cellular Integrity and Anthocyanin Content

**Figure 10:**
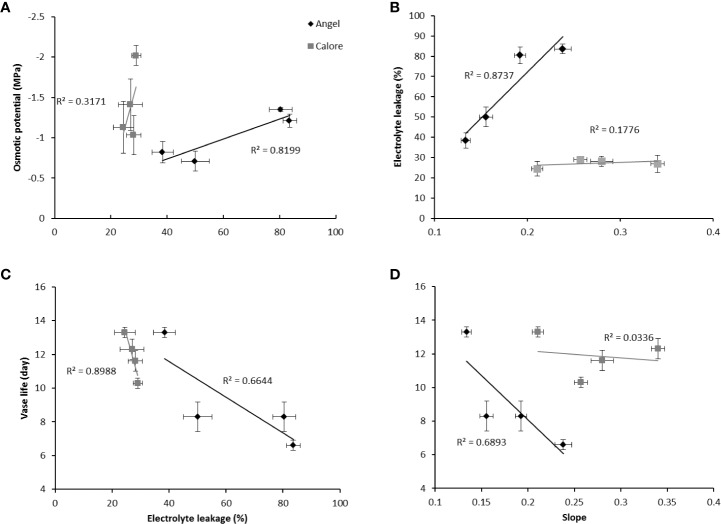
Relationships between electrolyte leakage (EL) and osmotic potential **(A)**, EL and slope of the curve for accumulative percentage of water loss over time **(B)**, EL and vase life **(C)** and vase life and slope of the curve for percentage of water loss over time **(D)** for the spathes of two cultivars of Anthurium cut flowers (‘Calore’ and ‘Angel’) after 14 days of exposure to different light spectra at 4°C. Values are the means of six biological replicates for the vase life and slope and three biological replicates for electrolyte leakage and osmotic potential ± SEM.

In ‘Angel’, after 14 days of light treatment, a positive relationship (R^2^ = 0.82) was found between the light effects on the osmotic potential and the EL of the spathe (**Figure 10A**). The EL of the spathe tissue was positively correlated with the slope of the water loss curve (R^2^ = 0.87) (**Figure 10B**). Both EL (R^2^ = 0.66) and the slope of the water loss curve (R^2^ = 0.68) were negatively correlated with the vase life (**Figures 10C, D**).

In contrast to ‘Angel’, in ‘Calore’, there were no clear relations between the above discussed parameters. Only a significant negative correlation was observed between the vase life and the EL (R^2^ = 0.89) (**Figure 10C**).

**Figure 11:**
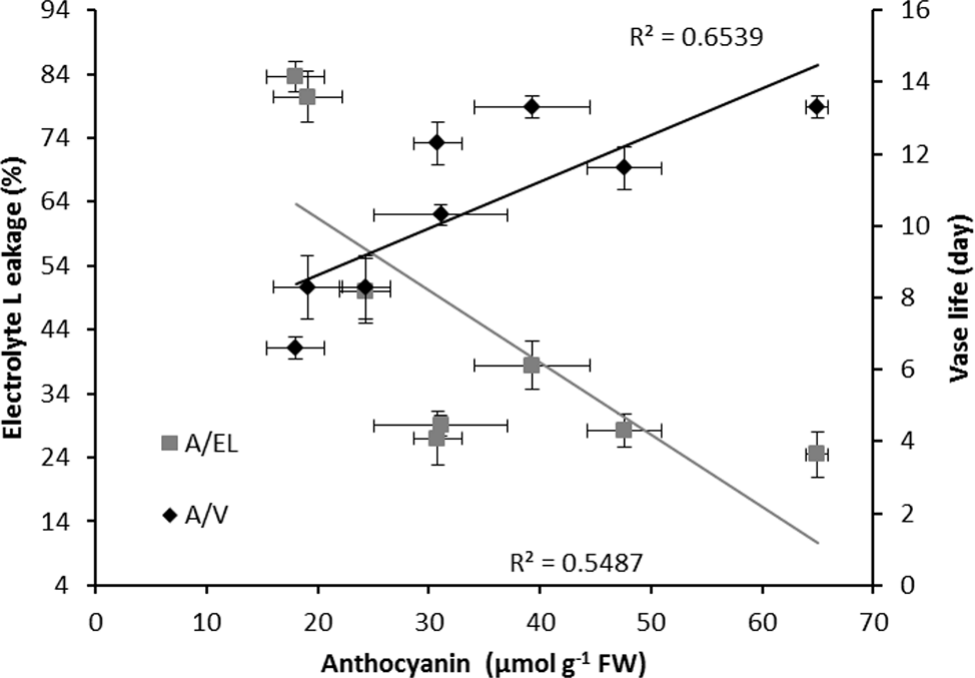
Relationships between anthocyanin content (A), electrolyte leakage (EL) and vase life (V) of Anthurium cut flowers (‘Calore’ and ‘Angel’) after 14 days exposure to different light spectra at 4^°^C. Values are the means of six biological replicates for the vase life and three biological replicates for electrolyte leakage and anthocyanin ± SEM.

In both cultivars, anthocyanin content of the spathe at day 14 was negatively correlated with EL (R^2^ = 0.55 for the combined data); anthocyanin was positively correlated with the vase life (R^2^ = 0.65 for the combined data) (**Figure 11**).

### Blue Light Is a Determinate Factor for the Vase Life of Anthurium at Chilling Temperature

**Figure 12:**
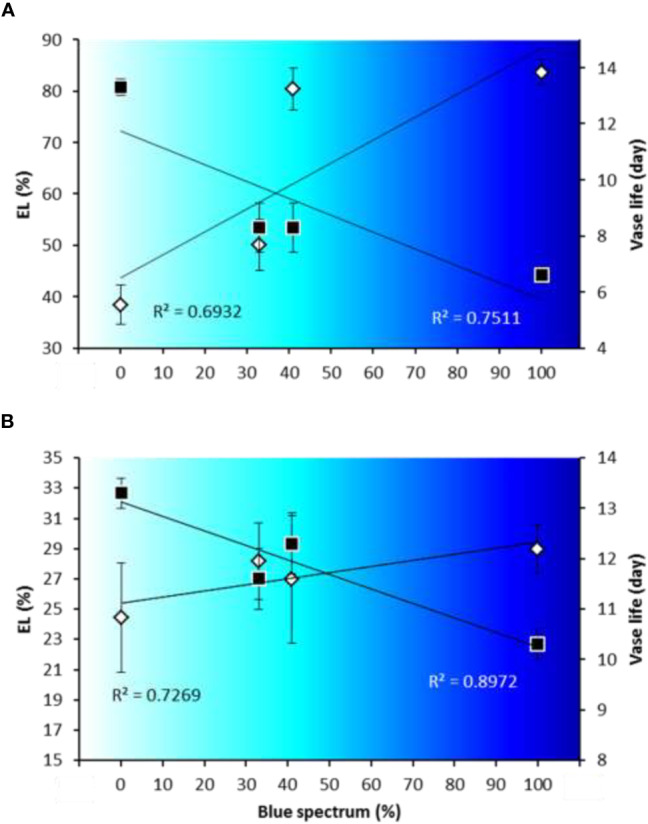
Relationships between percentage of blue light (400–500 nm) in overall spectral light composition [B spectrum (%)] and electrolyte leakage (EL) at day 14 and vase life for spathes of Anthurium cut flowers (‘Angel’ [panel **(A)**] and ‘Calore’ [panel **(B)**]) at 4°C. Gray symbols stand for electrolyte leakage (EL), and black symbols stand for vase life. Values are the means of six biological replicates for the vase life and three biological replicates for EL ± SEM.

The relation between vase life and EL depended on the percentage of B (400–500 nm) in the light spectrum (**Figure12**). A negative relationship was observed between the percentage of B in the light spectrum and the vase life in both Anthurium cultivars [R^2^ = 0.75 for ‘Angel’ (**Figure 12A**) and R^2^ = 0.89 for ‘Calore’ (**Figure 12B**)], and a positive relationship was found between the percentage of B in the light spectrum and EL [R^2^ = 0.69 for ‘Angel’ (**Figure 12A**) and R^2^ = 0.72 for ‘Calore’ (**Figure 12B**)].

## Discussion

Production of Anthurium cut flowers in cold seasons is challenged by the low temperatures during postharvest (Kamemoto, 1962). Cold storage is usually used to regulate supply of cut flowers (Faragher et al., 1984). However, exposure to cold negatively affects quality of Anthurium (Promyou and Ketsa, 2014).

Results obtained from current experiment showed that postharvest lighting is an important aspect determining the quality of Anthurium cut flowers during exposure to cold. In most of the studies on postharvest quality, the different light treatments were applied before harvest (Gruda, 2005; Reid and Jiang, 2012; Darko et al., 2014). As far as we are aware there is no reported study on the effects of postharvest light spectra on quality of cut flowers especially under cold storage.

In the present study we have used two cultivars differing in spathe color; ‘Calore’ with red and ‘Angel’ with white spathe. Overall, the response to the postharvest light treatments was comparable in the two cultivars. More B in the spectrum correlated with a shorter vase life and higher EL (**Figure 12**). Lower anthocyanin content was correlated with higher EL and shorter vase life (**Figure 11**), and more water loss was correlated with the higher EL and shorter vase life (**Figure 10**). The latter aspect, however, was evident in ‘Angel’ but not in ‘Calore’.

In the current study, postharvest exposure to B spectrum resulted in higher water loss from spathes of Anthurium especially when compared with R spectrum and D environment (**Figure 4**). Spathes of Anthurium contain stomata (Elibox and Umaharan, 2008), and it has been shown that spathe water content depends on its stomatal conductance (Farrell et al., 2012). B spectrum can affect different processes in plants including stomatal opening and photosynthesis (Tibbitts et al., 1983; Mao et al., 2005; Whitelam and Halliday, 2008), and a wide range of publications supported the role of the B spectrum in stimulating stomatal opening (Kinoshita et al., 2001; Talbott et al., 2002; Hayashi et al., 2011). Although promotion of stomatal opening has been reported by both B and R spectra, the effect of R spectrum on stomatal opening is indirect. B spectrum can directly induce ion influx into the guard cells and as a result promotes stomatal opening (Kinoshita and Hayashi, 2011), whereas the promoting role of R spectrum on stomatal opening is *via* its effects on mesophyll photosynthesis and guard cell chloroplasts (Olsen et al., 2002; Suetsugu et al., 2014).

Although many studies reported the importance of light intensity during postharvest storage of horticultural crops, there are not many reports regarding the effects of light spectrum on quality of horticultural products (Kasim and Kasim, 2007; Noichinda et al., 2007; Martínez-Sánchez et al., 2011; Witkowska, 2013; van Meeteren and Aliniaeifard, 2016). Many of these studies related the effects of light on postharvest quality to its effects on photosynthesis process (Toledo et al., 2003; Noichinda et al., 2007; Martínez-Sánchez et al., 2011). In the current study we did not detect any photosynthetic activity in the spathe of Anthurium (data not shown), which is probably due to lack of chlorophyll pigments in the spathe of these two Anthurium cultivars. The observed effects of light therefore cannot be attributed to photosynthetic processes. As a consequence, the R spectrum cannot promote stomatal opening, which led to lower water loss from Anthurium spathes in comparison with B-containing spectral compositions (B, W, and RB) (**Figures 5** and **6**). In the present study, measuring stomatal conductance was not attainable due to low temperature. However, water loss is often closely related to stomatal conductance.

At first glance, it may be speculated that B spectrum triggers stomata opening and thus promotes water loss and EL, which is not favorable for the vase life. Although this idea holds true for ‘Angel’, it did not apply to ‘Calore’ where water loss was not correlated with the EL and vase life (**Figure 10**). Woltering and Paillart (2018) studied the relationships between stomatal functionality and vase life of two rose cultivars during low temperature storage. They found that depending on the cultivar, stomatal functionality (closing response following dehydration) is associated with water stress and vase life of the rose cultivars (Woltering and Paillart, 2018). Similar results were obtained for ‘Angel’; however, no distinct correlation was found between water loss and vase life of ‘Calore’ (**Figure 10D**) indicating that other mechanisms are involved.

EL was correlated with osmotic potential (**Figure 10A**) and percentage of water loss (**Figure 10B**) in the spathe of Anthurium (especially for ‘Angel’). Water loss induces alterations in cellular metabolism (Wang et al., 2014), resulting in accumulation of soluble carbohydrate and proline (Chaudhuri et al., 2017). In our experiment, no correlation was found between water loss percentage and proline or soluble carbohydrate concentrations among different light spectra. This partial contradiction in our findings compared to the previous reports can be due to the impact of low temperature on induction of osmotic solutes (Li, 2018). Accumulation of different osmotic substances results in a decline in osmotic potential (Chaudhuri et al., 2017) and as a result membrane deterioration (Shibairo et al., 2002). Loss of membrane integrity increases EL from the tissues. The relationship between the rate of water loss and changes in EL indicates that water loss in Anthurium spathes is associated with membrane injury and a decline in vase life (**Figure 10C**). However, the relationship between the rate of water loss with EL in ‘Calore’ was not the same as in ‘Angel’, and it seems the vase life is controlled by other mechanisms than those regulated with water loss. Farrell et al. (2012) showed that different mechanisms are involved in determining vase life of different cultivars of Anthurium cut flowers, and they concluded that compared to Anthurium cultivars with long vase life, cultivars with a short vase life have higher stomatal conductance and lower spathe RWC. Although, ‘Angel’ is considered as a long vase life Anthurium cultivar (more than 40 days), its vase life is dramatically decreased by exposure to low temperature during storage (3–20 days) (Promyou et al., 2012; Ketrodsakul et al., 2016). Therefore, it can be considered as a short vase life cultivar under cold storage conditions. Our results in the case of ‘Angel’ are in accordance with Farrell et al. (2012), who showed that spathes water holding capacity affects EL and as a result vase life of Anthurium spathes. In the current study, negative relationships (R^2^ = 0.66 for ‘Angel’ and R^2^ = 0.89 for ‘Calore’) were detected between EL and vase life for both Anthurium cultivars (**Figure 10C**). Similarly, increase in EL due to low temperature exposure has been reported in spathes of different Anthurium cultivars (Farrell et al., 2012; Soleimani Aghdam et al., 2015; Soleimani Aghdam et al., 2016a).

ROS over-accumulation is one of the main reasons compromising cellular integrity (Mittler et al., 2004). However, no correlation was found between hydrogen peroxide levels and the vase life in Anthurium cultivars. ‘Calore’ with red spathe color had higher hydrogen peroxide content than ‘Angel’ under all studied light spectra (**Figure 8**), which further disapproves the involvement of hydrogen peroxide in determination of Anthurium vase life in cold storage.

Anthocyanins are plant pigments that usually accumulate in plant tissues in response to a wide range of stressors (Landi et al., 2015). These plant pigments can help plants to cope with light stress in two ways: by serving as a sun screen and through scavenging of free radicals (Landi et al., 2015). Apart from their sunscreen properties that restrict penetration of light into the plant tissues (Trojak and Skowron, 2017), anthocyanins through oxyradical scavenging activities can protect membranes and limit cell disruption (Takele, 2010; Zhang et al., 2012; Trojak and Skowron, 2017; Bayat et al., 2018). The ameliorative role of anthocyanins in different plant parts (*e.g.* leaf and fruit skin) in response to cold and light stresses has been reported (Sivankalyani et al., 2016; Bi et al., 2018). In Begonia leaves, elevation in transcript levels of the anthocyanin biosynthesis genes and as a consequence accumulation of anthocyanin was detected following six days of co-exposure to low temperature and light (300 μmol m^−2^ s^−1^), while no increase in transcript levels of the anthocyanin biosynthesis genes and anthocyanin level was detected in plants exposed to the same condition and treated with 3-(3,4-dichlorophenyl)-1,1-dimethylurea (inhibitor of light mediated anthocyanin accumulation) or in plants exposed to 25/15°C (day/night) and 300 μmol m^−2^ s^−1^ light intensity (Bi et al., 2018). These findings are indicative of the production of anthocyanins possibly in response to light under low temperatures as protectants against excessive ROS production and subsequent chilling injury. In agreement with these findings, a negative relationship between anthocyanin concentration and EL of the spathe and a positive relationship between anthocyanin concentration and vase life were observed in the both studied Anthurium cultivars (**Figure 11**). The protective role of anthocyanins on cellular integrity has been previously reported in different plant species following exposure to different abiotic stresses (Takele, 2010; Trojak and Skowron, 2017; Bayat et al., 2018). Furthermore, the protective role of anthocyanins on membrane integrity during co-exposure of plant leaves to light and low temperature stresses has been reported (Krol et al., 1995; Hoch et al., 2001). However, in our research no significant relationship between anthocyanin concentration and H_2_O_2_ in the spathe was observed in both studied cultivars. It has been shown that anthocyanins mainly accumulate in the vacuole and not in the chloroplasts where ROS accumulation occurs (Wise, 1995). This suggests that antioxidant activity is not the main function of anthocyanins during cold–light stress (Hoch et al., 2001). In our study, the anthocyanins were mainly detected in the vacuole of the epidermal cells (**Supplemental Figure 1**). Confirming this result we did not detect any chlorophyll and as a result no photosystem II activity in both Anthurium cultivars. Therefore, due to the absence of chloroplast, we can conclude that anthocyanin mainly accumulated in the vacuole. It has been reported that anthocyanin accumulation is a response to high ROS level in the leaves (Xu and Rothstein, 2018). High original H_2_O_2_ content (**Supplemental Figure 2**) in the spathe of ‘Calore’ can be a reason for high anthocyanin content for this cultivar (**Supplemental Figure 3**).

Our result showed that a higher percentage of B in the spectral light composition during postharvest phase resulted in higher EL and as a result shorter vase life in Anthurium cut flowers under cold storage conditions (**Figure 4**). In accordance with our results, Yu et al. (2017) reported that in *Camptotheca acuminata* B spectrum can cause damage to the membrane and an increase in EL, while R spectrum can prevent ROS accumulation in the cells which prevents electrolyte leakage from the cells.

**Figure 13:**
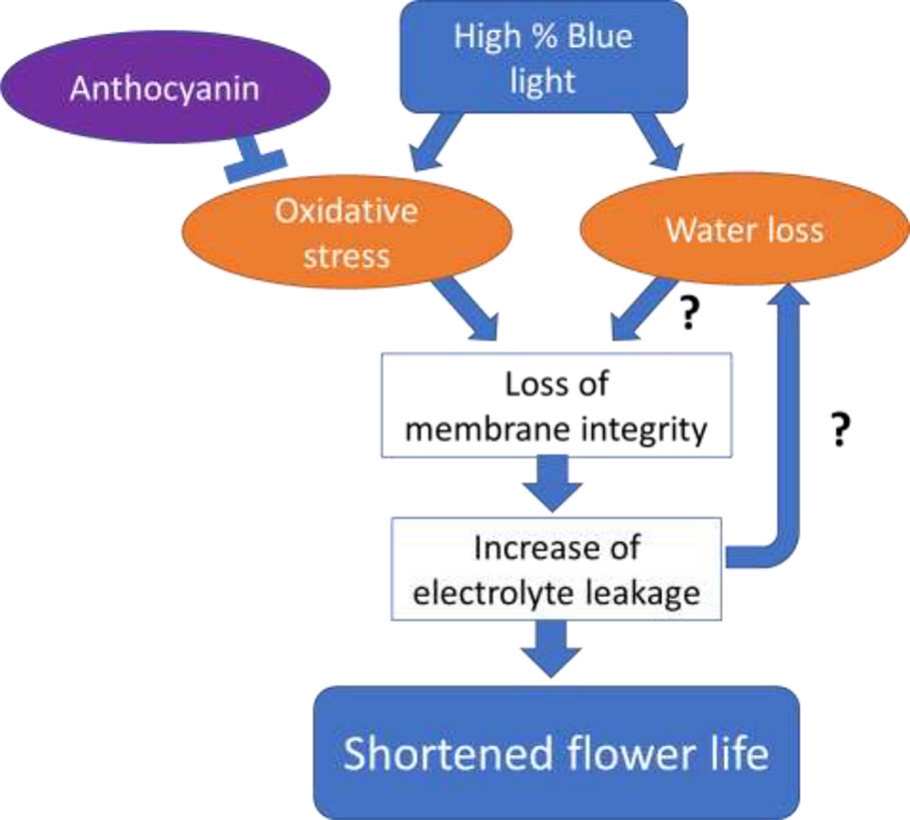
Hypothetical model for the effect of Blue light spectrum on Anthuriun vase life under chilling conditions. Original high ROS content causes accumulation of anthocyanins, but cold-induced oxidative stress breaks down anthocyanins. Anthocyanin may be degraded by Blue light but at the same time be used to neutralize the oxidative stress. Blue spectrum may affect stomatal conductance leading to water loss, changes in osmotic potential, decreasing membrane integrity and finally spathe senescence. Blue spectrum may at the same time induce oxidative stress, leading to loss of membrane integrity, electrolyte leakage and spathe senescence. Both routes may interact with each other.

The different processes that are associated with the senescence in the two different cultivars in response to the percentage of B in the spectrum may be explained by assuming a dual role of B spectrum in cold stored Anthurium flowers (**Figure 13**). Original high ROS content causes accumulation of anthocyanins, as it is occurred for the Calore cultivar. Anthocyanin may be degraded by the B light but at the same time be used to neutralize the oxidative stress. B spectrum may induce oxidative stress, indirectly through its negative effects on anthocyanin content or directly leading to loss of membrane integrity, electrolyte leakage, and senescence. In both cultivars there were significant correlations between the vase life and ion leakage and anthocyanin. This indicates that this route is important in explaining the effect of B light. The magnitude of the effect of B light was less in ‘Calore’ than in ‘Angel’; this may be related to the higher amount of anthocyanin naturally present in the spathe of ‘Calore’ (**Supplemental Figure 3**). On the other hand, the B spectrum may affect stomatal conductance leading to increased water loss, changes in osmotic potential and as a result loss of membrane integrity and senescence. Both routes may interact with each other.

In ‘Angel’, in addition to the oxidative stress route, there were also good correlations between the vase life and water loss, which was much less in the case of ‘Calore’. This is caused by the relation between the percentage of the B light spectrum and the water loss. In ‘Angel’, water loss shows a linear relation with the percentage of the B light spectrum, indicating that stomatal conductance increases with increasing B spectrum; in ‘Calore’, however, maximum water loss occurs in 40% of B in the spectrum (**Figure 4**). The observations that in both cultivars there are good correlations between the vase life and the factors of the oxidative stress route indicate that the oxidative stress route is more important than the water loss route for the explanation of the effect of B light under these specific conditions (lighting in the cold condition).

This study is the first report showing the importance of light quality on determination of vase life of Anthurium under cold temperatures. However, further investigations regarding the effects of different temperatures, different intensities of light spectra and a wide range of Anthurium cultivars are needed to reach a practical solution for low temperature storage of cut Anthurium.

## Conclusion

Anthurium is a tropical cold sensitive plant, which needs to be stored at 12.5–20°C. In the winter time and in cold environments, chilling injuries to Anthurium spathes decrease its marketability. Here, we showed that the spectral light composition during storage of Anthurium cut flowers is an important environmental factor determining postharvest flower performance. A high percentage of B in the light spectrum was associated with increased EL of the spathe and a shorter vase life. Therefore, postharvest handling of Anthurium cut flowers should preferably be performed in an environment with limited B spectrum when exposures to low temperatures are inevitable.

## Data Availability Statement

All datasets generated for this study are included in the article/**Supplementary Material**.

## Author Contributions

SA made substantial contributions to conception and design, also performed statistical analysis, drafted the manuscript, and critically revised the final version. ZF carried out the experiments, collected and critically analyzed the scientific literature, and helped in the writing of the manuscript. SD took part in designing and planning the experiments, preparing of material for the experiment. TL helped in the preparation of material for the experiment and took part in designing and planning of the experiments and critical revision of the final manuscript. EW contributed to conception and design of the final manuscript, scientific discussion, and critical revision of the final manuscript.

## Conflict of Interest

The authors declare that the research was conducted in the absence of any commercial or financial relationships that could be construed as a potential conflict of interest.
