# Supplementary material for: Postharvest Spectral Light Composition Affects Chilling Injury in Anthurium Cut Flowers
Source: Front Plant Sci. 2020 Jun 12;11:846. doi: 10.3389/fpls.2020.00846 (PMC7304073; doi:10.3389/fpls.2020.00846)
Supplement: Supplementary file 1 [file Presentation_1.pdf]

Supplemental Figures

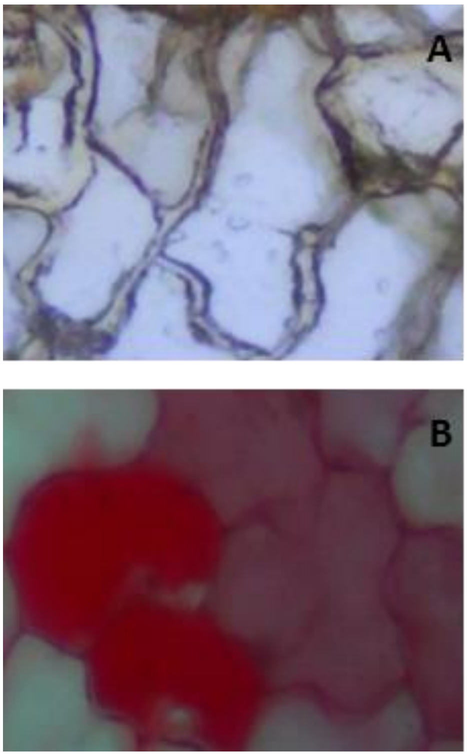

Supplemental Figure 1. Lack of pigmentation in Angel cultivar (A) and accumulation of anthocyanin in Calore cultivar (B) in Anthurium spathe

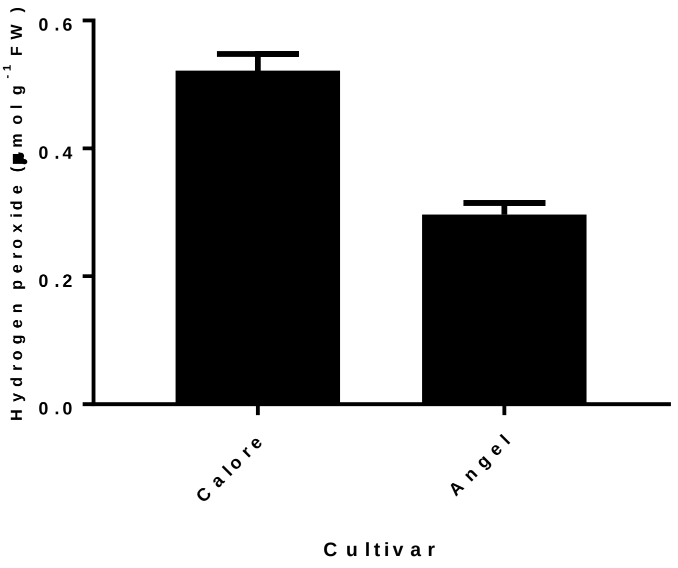

Supplemental Figure 2. Hydrogen peroxide content in the spathes of two cultivars of Anthurium cut flowers (‘Calore’ and ‘Angel’). Values are the means of fifteen biological replicates and columns indicate means ± SEM.

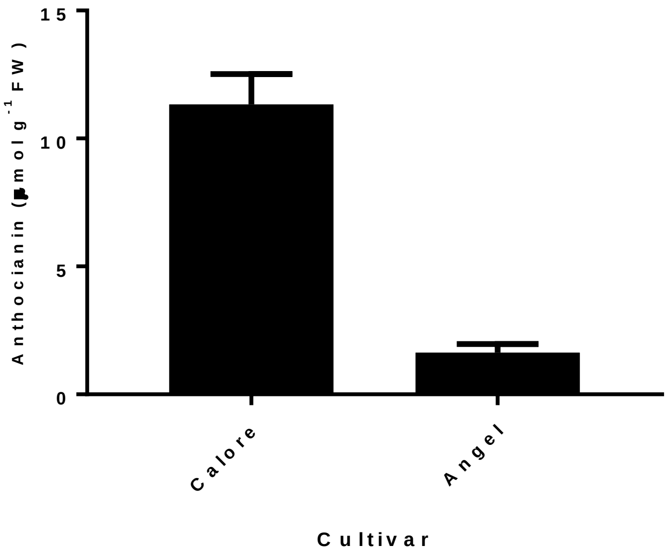

Supplemental Figure 3. Anthocyanin content in the spathes of two cultivars of Anthurium cut flowers (‘Calore’ and ‘Angel’). Values are the means of fifteen biological replicates and columns indicate means ± SEM.
